# First steps in identifying and addressing loneliness in the context of COVID-19

**DOI:** 10.1177/1757913920975793

**Published:** 2021-02-25

**Authors:** KB Cunningham, T Kroll, M Wells

**Affiliations:** Dr, Division of Population and Behavioural Sciences, School of Medicine, University of St Andrews, North Haugh, St Andrews, Scotland, KY16 9TF, UK; Professor, UCD Centre for Interdisciplinary Research, Education and Innovation in Health Systems (UCD IRIS), UCD School of Nursing, Midwifery and Health Systems, UCD Health Sciences Centre, University College Dublin (UCD), Dublin, Ireland; Professor, Nursing Directorate, Imperial College Healthcare NHS Trust, Charing Cross Hospital, London, UK

*Already a significant public health problem prior to the COVID-19 pandemic due to its associations with low wellbeing, poor mental and physical health and premature mortality, loneliness has likely escalated greatly across the world following the introduction of physical distancing measures to control the spread of coronavirus. In this article, Kathryn Cunningham and co-authors outline how addressing loneliness and its related adverse health outcomes is therefore critical in the longer-term response to the pandemic.*

*Coronavirus and social distancing has forced all of us to look loneliness in the eye.* ^1^

Prior to the COVID-19 pandemic, loneliness was already a serious public health problem in Western society, due to both its prevalence and its associations with low wellbeing, poor mental and physical health, and premature mortality.^2–4^ The relevance and significance of the problem has been amplified by the introduction of physical distancing measures to control the spread of coronavirus. Such measures have changed the nature of people’s interaction and communication with others and have raised considerable international concern regarding their adverse impact on loneliness.^5–7^ This concern has been validated by evidence that loneliness has increased in several countries during the COVID-19 pandemic.^8–11^ Addressing loneliness, and its associated health outcomes, has been identified as critical in the long-term response to the pandemic.^12^

Loneliness is challenging to address due to its multidimensional nature and the resulting need for tailored interventions that target the root causes of the problem.^13,14^ This challenge is compounded by both the lack of a unified definition of loneliness and the absence of standardised measures to identify loneliness.^3,13^

Based on the findings of our theoretical concept analysis and qualitative study of loneliness (see Cunningham et al.^15^ for an overview of these) and a decade of discussions with academics, health, social care and third-sector professionals, patients and the public, we present a comprehensive conceptualisation and definition of loneliness. These facilitate understanding of the problem and provide a basis for effective identification, assessment and intervention. We use them to illustrate how COVID-19-related physical distancing measures might generate or exacerbate loneliness. We then suggest potential interventions for different experiences of loneliness in the context of COVID-19.

We have identified that loneliness comprises social and emotional elements and its root causes are self-perceived deficiencies in four types of relationships: (1) emotional (e.g. intimate partner); (2) social (e.g. engaging friendship); (3) cultural (e.g. group affiliation); and (4) professional (e.g. supportive healthcare). It can therefore be defined as ‘*the negative feeling a person experiences when the quantity or quality of his/her emotional, social, cultural or professional relationships does not meet what (s)he needs or desires*’.

COVID-19-related physical distancing measures may adversely affect all four types of relationships, thereby generating or exacerbating loneliness. For example, the forced confinement of families and the inability to be physically close to a partner or spouse residing separately may strain or even damage emotional relationships, while the disruption of normal in-person activities such as meeting a friend for a face-to-face coffee catch-up might impair social relationships. The cessation of group meetings such as religious services and the requirement to work from home may reduce a sense of belonging, therefore weakening cultural relationships, and the necessity of personal protective equipment (PPE) for staff and the postponement of investigations and treatments might compromise supportive healthcare relationships.

Given that different people’s loneliness experiences stem from different self-perceived relationship deficiencies, a ‘one size fits all’ approach to preventing or mitigating loneliness in the context of COVID-19 is unlikely to be appropriate. Instead, tailored interventions that target the self-perceived relationship deficiencies generating or exacerbating different loneliness experiences (i.e. the root causes of the problem) are necessary. For example, an effective intervention for a person whose loneliness stems from a strained relationship with his or her partner due to forced confinement might include a range of strategies to explore expectations and rebuild communication and intimacy. However, loneliness that arises from the disruption of a weekly face-to-face coffee catch-up with a friend is likely to need a different approach – perhaps a weekly telephone catch-up with that friend could help to alleviate those feelings of loneliness. Loneliness emanating from a reduced sense of belonging due to cessation of normal religious services is likely to need yet another approach, such as connection with an online community of others with similar religious beliefs and participation in virtual religious services, while an effective intervention for a person feeling lonely due to postponement of treatment for a health condition might include having questions and concerns addressed by a trusted healthcare professional.

Addressing loneliness in the context of COVID-19 therefore requires an understanding of the complexity of the problem. Our conceptualisation and definition facilitate identification and assessment of loneliness, promoting consideration of which relationships are adversely affected by physical distancing measures, how, and why. This enables signposting to, or development of, tailored interventions to prevent or alleviate loneliness by targeting the root causes of the problem, thereby reducing loneliness and its related adverse health outcomes.
